# Peridynamics analysis of crack propagation in concrete considering random aggregate distribution

**DOI:** 10.1038/s41598-025-87582-8

**Published:** 2025-02-04

**Authors:** Bin Geng, Ze Li, Yigong Zhao, Xiaoyan Zhang

**Affiliations:** 1 Faculty of Civil Engineering and Mechanics, Kunming University of Science and Technology, Kunming, 650500 Yunnan China; 2 Faculty of Electric Power Engineering, Kunming University of Science and Technology, Kunming, 650500 Yunnan China

**Keywords:** Peridynamics, Random aggregates, Numerical simulation, Monte Carlo simulation, Crack propagation, Engineering, Materials science

## Abstract

The mechanical properties and fracture behavior of concrete are controlled by aggregate characteristics, and the distribution of aggregates is uncertain. Traditional studies on concrete crack propagation mainly conduct deterministic analysis based on the position and size of the aggregates, rarely considering the uncertainty of aggregate distribution. Based on the Peridynamics (PD) theory, random distribution functions are introduced to describe the geometric characteristics and positional parameters of concrete aggregates. Simulating the effect of random distribution of aggregates on concrete crack propagation by presetting random aggregates. For the first time, the Boundary Damage Ratio (BDR) is proposed to quantitatively describe the influence of cement mortar and aggregate on crack propagation, revealing the influence rules of random aggregate parameters on concrete damage provides a new method for studying concrete crack propagation. The research results show that the size and position of aggregates determine the crack propagation path during concrete failure. The BDR can indicate the quality of the concrete grading and the intensity of the aggregate’s guiding effect on crack propagation. It was found that the aggregate size and the BDR follow a Weibull distribution; the larger the aggregate size, the smaller the shape parameter.

## Introduction

The presence of cracks reduces the integrity of concrete structures, leading to a degradation of material performance. Aggregates, as a crucial component of concrete mix, form the skeleton of the concrete^1–3^. During the manufacturing and service life of concrete materials, the size, position, and mechanical properties of aggregates introduce significant uncertainties in the crack propagation process. Therefore, studying the initiation and propagation of cracks in concrete under random aggregate conditions holds significant engineering practical importance. Exploring the influence of cement mortar and aggregates on the crack propagation in concrete is a crucial issue in the field of concrete research.

Conventional analysis methodologies predominantly employ the finite element method (FEM)^4,5^ or extended finite element method (XFEM)^6,7^ to simulate the crack expansion of concrete. The FEM, as a simulation method based on the theory of continuous medium mechanics, requires constant mesh reconstruction during the crack extension process of the concrete model. The meshless method^8^ eliminates this mesh dependence, but it is difficult to use it as a desirable method to simulate crack extension due to the fact that its higher-order continuum approximation function nature is not applicable to solving the discontinuity problem when in the crack extension condition^9^. Extended finite element method, cohesive zone model (CZM)^10^ and lattice model^11^ simulation methods are also commonly used in the numerical simulation of concrete models. However, the theoretical basis of the above methods is based on the spatial partial differential equations and continuity assumptions of the traditional theory of fracture mechanics. Cracks lead to discontinuities in the displacement field at the tip of the cracks, the partial derivatives do not exist, and the spatial derivatives of the partial differential equations lose their significance.

In 2000, Dr. Silling from the Sandia National Laboratories in the United States proposed the PD theory^12^. In 2010, Huang, Zhang, et al.^13^ introduced it to China. The early PD theory was named "bond-based peridynamics." In 2007, Silling introduced more general equations to create "state-based peridynamics"^14^. PD theory, based on its unique non-local numerical calculation methods, solves spatial integral equations to describe the forces on material points without requiring displacement derivatives. This makes it well-suited for addressing discontinuities, overcoming the limitations of traditional methods in dealing with such issues, and offering inherent advantages in simulating crack propagation^15–19^.

Due to the inherent advantages of PD theory in studying crack propagation, an increasing number of scholars have used PD theory to investigate the crack propagation process in concrete in recent years. Yang, Dong, et al.^20^ proposed a new damage model within the bond-based peridynamics (BBPD) framework to quantitatively study type I crack propagation in concrete. Huang, Kong, et al.^21^ conducted numerical studies on the dynamic tensile failure of concrete, proposing a PD-based model for this phenomenon. These research achievements have provided new methods for exploring the crack propagation process in concrete using PD theory but focused only on specific types of concrete without considering the role of aggregates in practical engineering. Wu, Huang, et al.^17^ used PD theory to simulate the dynamic fracture and failure of three-dimensional concrete structures. Zhang, Hou, and others^22^ studied the macroscopic mechanical properties and fracture processes of concrete under uniaxial tension. The research outcomes of Wu, Zhang, et al.^23^ incorporated the effects of cement mortar, aggregates, and the interfacial transition zone (ITZ) in the concrete model, aligning more closely with actual engineering concrete. However, considering the uncertainty and unpredictability of aggregate distribution in concrete, adopting non-deterministic analytical methods and computational approaches is necessary.

In conclusion, this paper considers the randomness of aggregate gradation, geometric parameters, and positions, and conducts numerical simulations of concrete fracture failure based on the Monte Carlo method. It introduces the BDR to describe the impact of aggregates on cement mortar during concrete failure, revealing the influence of aggregates on the macroscopic crack propagation of concrete.

## Peridynamics theory

### Basic principles of Peridynamics

In 2000, Silling et al.^14^ proposed the non-local Peridynamics (PD) theory to address the discontinuity issues inherent in both classical local and non-local continuum mechanics theories. Compared to classical continuum mechanics, PD is more suitable for describing fracture and material behavior, especially in the presence of significant cracks or damage.

**Fig. 1:**
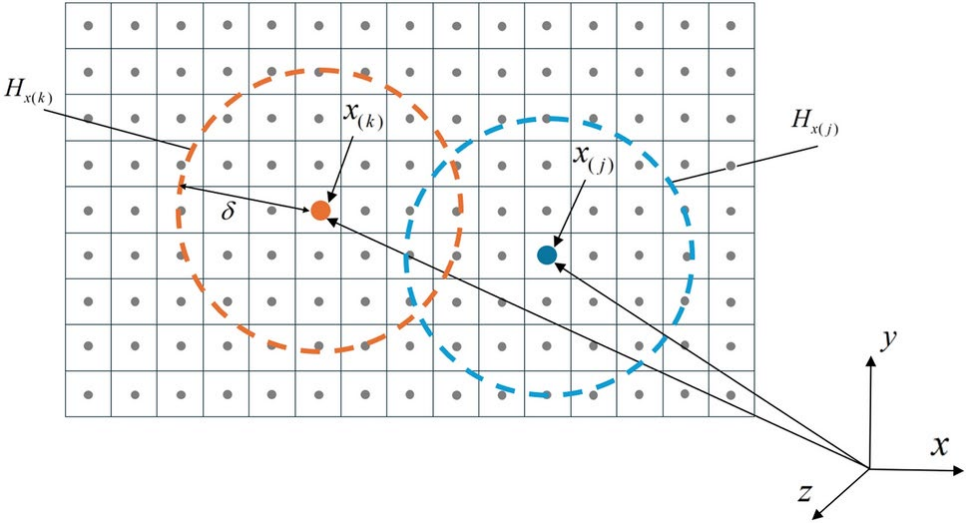
Interaction among PD particles.

Classical continuum mechanics theory, as a local theory, only assumes that a given material point interacts solely with its directly adjacent material points. The PD equation uses an integral equation form, allowing damage to occur at multiple locations within the material without the need for specific crack propagation criteria, and it can propagate along any path. The PD theory studies the physical phenomena involving the interactions between a material point and all other material points within its range of influence. As shown in Fig. 1, the influence range of a particle $x_{(k)}$ is defined by the horizon $\delta$. The $x_{(k)}$ within the $\delta$ of $x_{(k)}$ is referred to as its family $H_{{x_{(k)} }}$. The interaction between material points is controlled by the micropotential energy (a function of the deformation and the material’s intrinsic properties), and the localization of the interaction depends on the size of the horizon $\delta$. The smaller $\delta$ is, the more localized the interaction is. Therefore, the classical elasticity theory can be regarded as the limiting case of the PD theory where the near-field range tends to zero^23^.

In classical continuum mechanics, the equations governing the motion of a material point or cell in the computational domain are usually expressed as^23^:

1 $$\rho {\mathbf{\ddot{u}}}({\mathbf{x}}) = \nabla \cdot \sigma ({\mathbf{x}}) + {\mathbf{b}}({\mathbf{x}})$$

where: $\rho$ is the material density;${\mathbf{u}}$ is the displacement vector;${\mathbf{b}}$ is the body force vector.

**Fig. 2:**
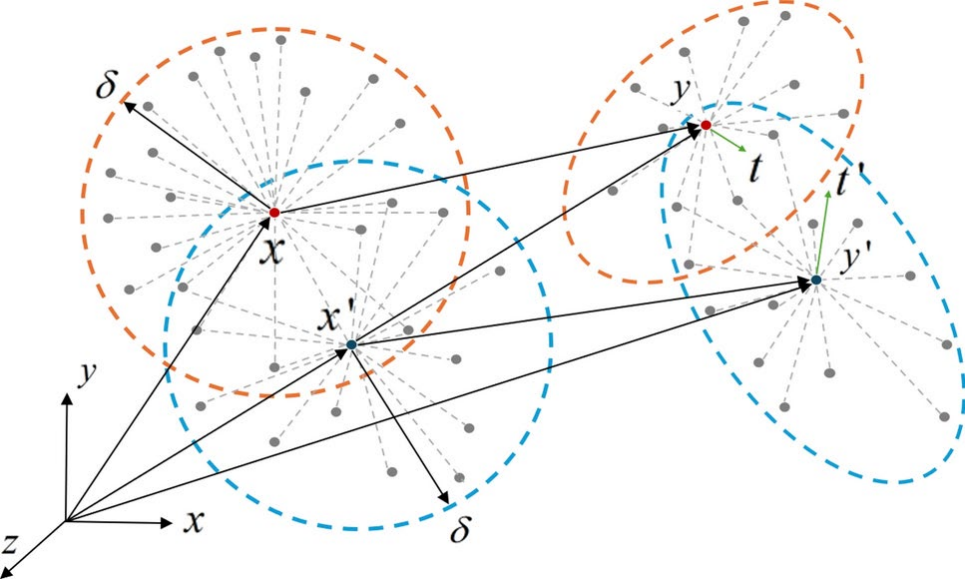
Deformation of PD particles $x$ and $x^{\prime}$ and the resulting paired force density vectors of equal magnitude and opposite directions.

The fundamental expression for the motion equations of the PD theory model is given by^23^:

2 $$\rho ({\mathbf{x}}){\mathbf{\ddot{u}}}({\mathbf{x}},t) = \int\limits_{H} {[{\mathbf{\rm T}}({\mathbf{u^{\prime}}} - {\mathbf{u}},{\mathbf{x^{\prime}}} - {\mathbf{x}},t) - {\mathbf{T^{\prime}}}({\mathbf{u}} - {\mathbf{u^{\prime}}},{\mathbf{x}} - {\mathbf{x^{\prime}}},t)]{\text{dH}} + {\mathbf{b}}({\mathbf{x}},t)}$$

where:$\rho$ is the material mass density; ${\varvec{u}}$ is the displacement vector of particle $x$;${\mathbf{u^{\prime}}}$ is the displacement vector of particle $x^{\prime}$;${\mathbf{b}}$ is the body force density of external loads; ${\mathbf{T}}$ is the force density vector of particle $x$; ${\mathbf{T^{\prime}}}$ is the force density vector of particle $x^{\prime}$;

The force density vector ${\mathbf{T}}$ represents the interaction forces between particles in the bond-based PD model. The force density vectors can be equal in magnitude and parallel in direction to the relative site vectors in the deformed configuration, as shown in Fig. 2, to satisfy the law of conservation of angular momentum.

This can be expressed as^23^:

3 $${\mathbf{T}}({\mathbf{u^{\prime}}} - {\mathbf{u}},{\mathbf{x^{\prime}}} - {\mathbf{x}},t) = \frac{1}{2}C\frac{{{\mathbf{y^{\prime}}} - {\mathbf{y}}}}{{\left| {{\mathbf{y^{\prime}}} - {\mathbf{y}}} \right|}}$$

Where:${\mathbf{y^{\prime}}} - {\mathbf{y}}$ is the relative displacement;$C$ is an undetermined auxiliary parameter.

Combining Eqs. (2) and (3), we can derive the bond-based PD motion equation for particle $x$^23^:

4 $$\rho ({\mathbf{x}}){\mathbf{\ddot{u}}}({\mathbf{x}},t) = \int\limits_{H} {f({\mathbf{u^{\prime}}} - {\mathbf{u}},{\mathbf{x^{\prime}}} - {\mathbf{x}},t)dH + {\mathbf{b}}({\mathbf{x}},t)}$$

where: $f({\mathbf{u^{\prime}}} - {\mathbf{u}},{\mathbf{x^{\prime}}} - {\mathbf{x}})$ is the point force response function, representing the force vector exerted by particle $x^{\prime}$ on particle $x$ per unit volume squared. Using ${{\varvec{\upmu}}}$ to represent the relative displacement ${\mathbf{u^{\prime}}} - {\mathbf{u}}$ between particles, and ${{\varvec{\upxi}}}$ to represent the relative position ${\mathbf{x^{\prime}}} - {\mathbf{x}}$, we obtain^23^:

5 $$f({{\varvec{\upeta}}},{{\varvec{\upxi}}}) = \frac{{{{\varvec{\upeta}}} + {{\varvec{\upxi}}}}}{{\left| {{{\varvec{\upeta}}} + {{\varvec{\upxi}}}} \right|}}cs\mu (t,{{\varvec{\upxi}}})$$

where:$c$ is the micro-modulus function, for one-dimensional problems: $c = 2E/A\delta^{2}$; for two-dimensional plane strain problems: $c = 12E/\pi \delta^{3} (1 + v)$;for two-dimensional plane stress problems: $c = 6E/\pi \delta^{3} (1 - v)$; for three-dimensional problems: $c = 12E/\pi \mu^{4}$;$s$ is the relative elongation rate between particles; $E$ is the tensile elastic modulus;$v$ is the Poisson’s ratio.

During the PD solving process, it is necessary to compute the displacement of each particle and the elongation rate $s_{0}$ between each pair of particles. When the elongation rate between a pair of particles exceeds the critical elongation rate, damage occurs. At this point, the value of the time-history scalar function $\mu$ is set to 0. Consequently, the corresponding interaction force density vector also disappears, and no force will act between them anymore. This process can be described using a scalar-valued function $\mu ({{\varvec{\upeta}}},{{\varvec{\upxi}}},t)$^23^:

6 $$\mu ({{\varvec{\upeta}}},{{\varvec{\upxi}}},t) = \left\{ {\begin{array}{*{20}l} {1,} \hfill & {s < s_{0} } \hfill \\ {0,} \hfill & {{\text{other}}} \hfill \\ \end{array} } \right.$$

The scalar-valued function $s_{0}$ represents the critical elongation rate of the "bond," which is related to the fracture energy release rate in classical linear elastic fracture mechanics. $s_{0}$ can be expressed in PD as^23^:

7 $$s_{0} = \left\{ \begin{gathered} \sqrt {\frac{4\pi G}{{9E\delta }}} ,\;{\text{for two - dimensional problems}} \hfill \\ \sqrt {\frac{5G}{{6E\delta }}} ,\;{\text{for three - dimensional problems}} \hfill \\ \end{gathered} \right.$$

where:$G$ is the material fracture energy release rate;$E$ is the elastic modulus;$\delta$ is the neighborhood radius.

Silling, Askari, et al.^23^ defined PD damage as the ratio of the number of broken bonds to the total number of bonds in the horizon $\delta$ of the material point $\varphi (x,t)$ to describe the crack extension path with the expression:

8 $$\varphi (x,t) = 1 - \frac{{\int\limits_{H} {\mu ({\mathbf{x^{\prime}}} - {\mathbf{x}},t)dV^{\prime}} }}{{\int\limits_{H} {dV^{\prime}} }}$$

where: $0 \le \varphi \le 1$,$\varphi$ = 0 indicates no damage occurring to the material point, and $\varphi$ = 1 indicates complete damage to that material point.

### Numerical methods

The PD theory postulates that a family $H_{{x_{(k)} }}$ of a particular material point is composed of countless material points, each possessing fundamental physical properties. For computational purposes, the material can be discretized as finite, uniformly shaped, and regularly arranged cubes. The length of the cube’s edge is denoted by $\Delta x$, and the volume of the cube is denoted by $(\Delta x)^{3}$. These small cubes combine to form the basic structural model of the analyzed object. Compared to finite element methods, this discretization approach does not require complex meshing like finite element methods do. The resulting structure is simple and regular^23^.

The motion equations of the Peridynamics theory, considering the interactions between material point ${x}_{i}$ and other material points within its neighborhood $\delta$, can be expressed in a discrete form^23^:

9 $${{\varvec{\uprho}}}_{{\mathbf{i}}} {\mathbf{u}}_{i} = \sum\limits_{j} {f({\mathbf{u}}_{{\mathbf{j}}}^{{\mathbf{n}}} - {\mathbf{u}}_{{\mathbf{i}}}^{{\mathbf{n}}} ,{\mathbf{x}}_{{\mathbf{j}}} - {\mathbf{x}}_{{\mathbf{i}}} )V_{j} + {\mathbf{b}}_{{\mathbf{i}}}^{{\mathbf{n}}} }$$

where:$n$ is the time step number; ${{\varvec{\uprho}}}_{{\mathbf{i}}}$ is the mass density of material point $i$;$u_{j}^{n}$ is the displacement of material point $x_{i}$ at time step $n$ $V_{j}$ is the volume of the cube at material point $j$;$b_{i}^{n}$ is the body force density exerted on material point $x_{i}$ at time step $n$.

Material points at the domain boundary contain portions that are both within and outside the horizon $\delta$. Therefore, a volume correction factor is introduced. The volume of material point $j$ is represented as^23^:

10 $$V_{j} = \left\{ {\begin{array}{*{20}l} {(\Delta x)^{3} ,} \hfill & {\left| {{\varvec{\upxi}}} \right| \le \delta - r} \hfill \\ {\frac{{\delta + r - \left| {{\varvec{\upxi}}} \right|}}{2r}(\Delta x)^{3} ,} \hfill & {\delta - r < \left| {{\varvec{\upxi}}} \right| \le \delta } \hfill \\ {0,} \hfill & {{\text{other}}} \hfill \\ \end{array} } \right.$$

In numerical simulation computations, it is necessary to set up boundaries as virtual boundary layers to impose different velocity boundary conditions. Therefore, setting boundary $3\Delta x$ as a virtual boundary layer, the time step $\Delta t$ satisfies the stability requirement according to the following equation ^23^:

11 $$\Delta t < \sqrt {\frac{2\rho }{{\sum {V_{k} \left| {C(x_{k} - x_{i} )} \right|} }}}$$

where: $V_{k}$ is the volume at material point $k$;$C$ is the stiffness matrix.

## Establishment of random aggregates concrete model

There are various scales of aggregates within concrete, and existing analysis methods cannot fully encompass and predict this uncertainty. Therefore, based on the PD theory and the Monte Carlo statistical principle, this paper adopts a large-sample Monte Carlo random simulation method. By randomly generating aggregate models under different gradation conditions, it conducts large-sample PD simulations of concrete structures. This approach intuitively reproduces the failure paths of concrete under the uncertainty of aggregate size and distribution. Additionally, it establishes the BDR to statistically characterize concrete fracture and failure, achieving a transition from deterministic to non-deterministic analysis of concrete crack propagation.

### Model establishment

**Fig. 3:**
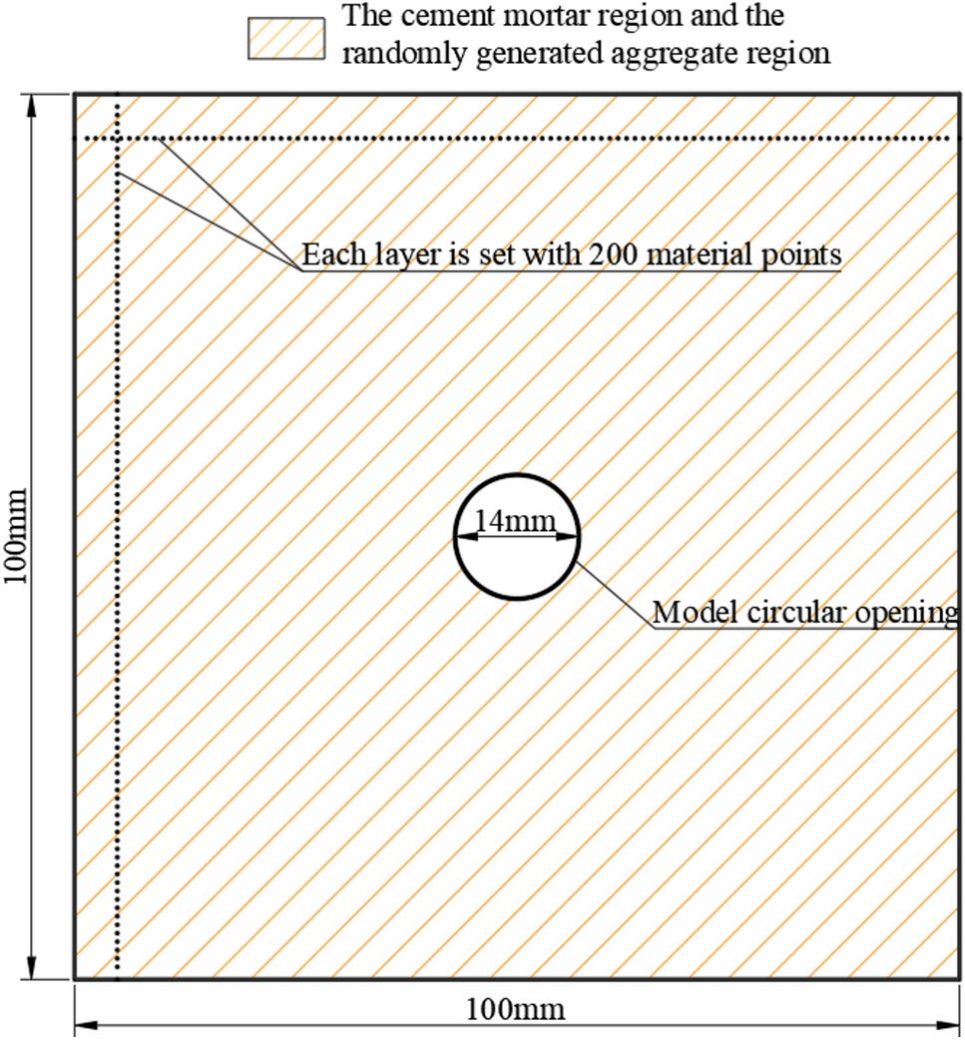
Model Establishment.

The 2D computational model of random aggregate concrete in this paper is shown in Fig. 3. The model has a length and width of 100 mm, with a pre-set circular opening of 7 mm radius at the center. The model is discretized into 200 material points along both the horizontal and vertical directions, excluding the circular opening at the center, resulting in a final model with a total of 40,584 material points.

### Random aggregate model parameter definition

**Fig. 4:**
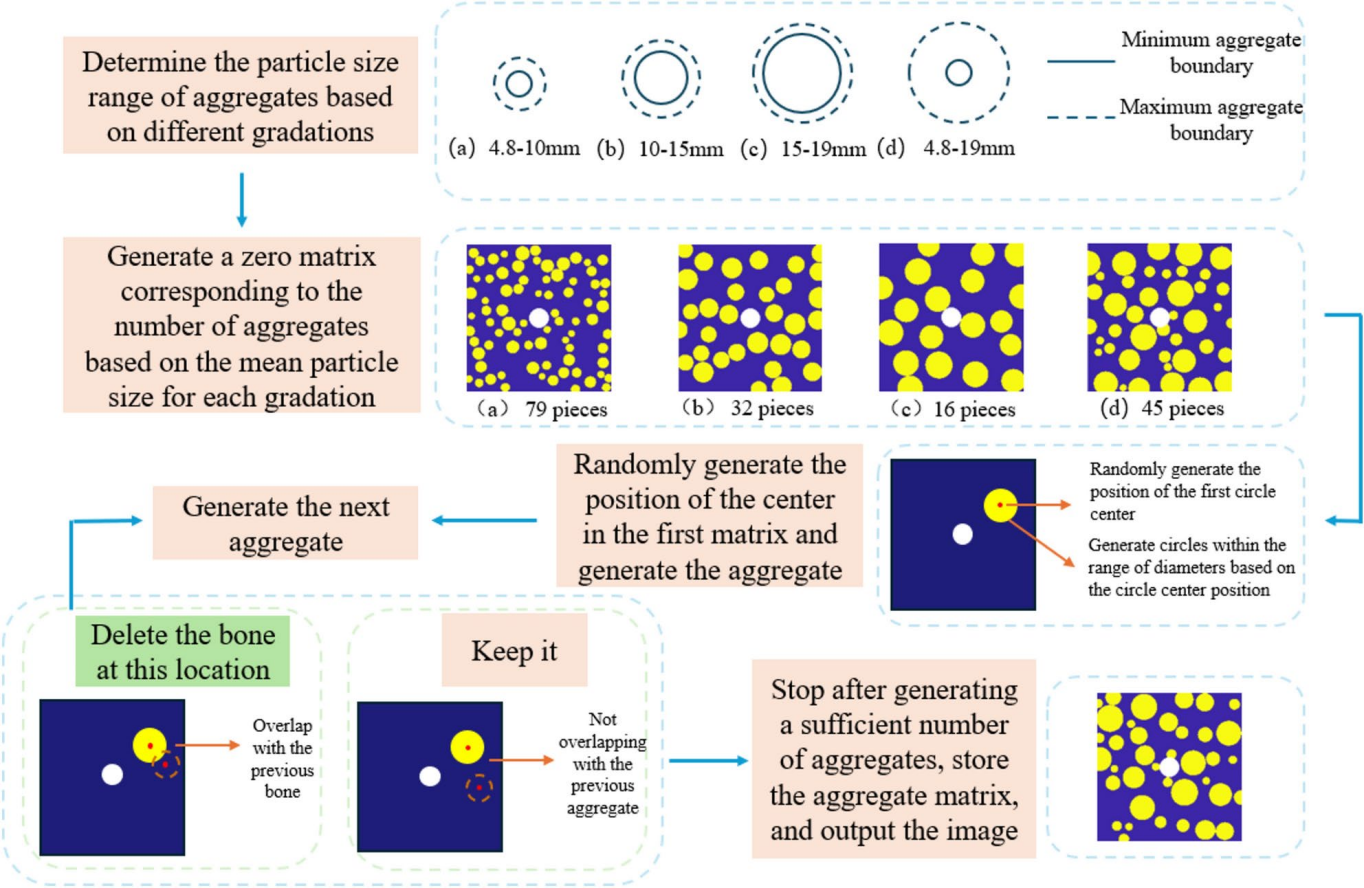
Schematic Diagram of Random Aggregate Model.

In the model, any material point $x_{(k)}$ has three possible relationships with other material points in its family $H_{{x_{(k)} }}$: (1) cement mortar to cement mortar; (2) cement mortar to aggregate; (3) aggregate to aggregate. Therefore, the mechanical parameters of three materials need to be defined: cement mortar, aggregate, and the interface between cement mortar and aggregate. The parameter definitions^24^ for the random aggregate model are shown in Table 1. In this paper, the random aggregate concrete model considers only coarse aggregates, which account for approximately 40% of the overall model, while fine aggregates are negligible and can be considered as the same material as cement mortar. The coarse aggregates have a minimum particle size of 4.8 mm and a maximum particle size of 19 mm. Three gradations are designed: 4.8–10 mm, 10–15 mm, 15–19 mm, and a control group with unfavorable gradation of 4.8–19 mm. The random aggregate generation process is shown in Fig. 4.

**Table 1:** Mechanical Parameters of the Model.

Material type	Elastic modulus (GPa)	Critical elongation ($s_{0}$)	Poisson’s ratio	Density (kg/m^3^)
Cement mortar	27.8	0.02	1/3	1500
Aggregate	55.3	0.04	1/3	2500
Interface	25	0.01	1/3	1200

**Fig. 5:**
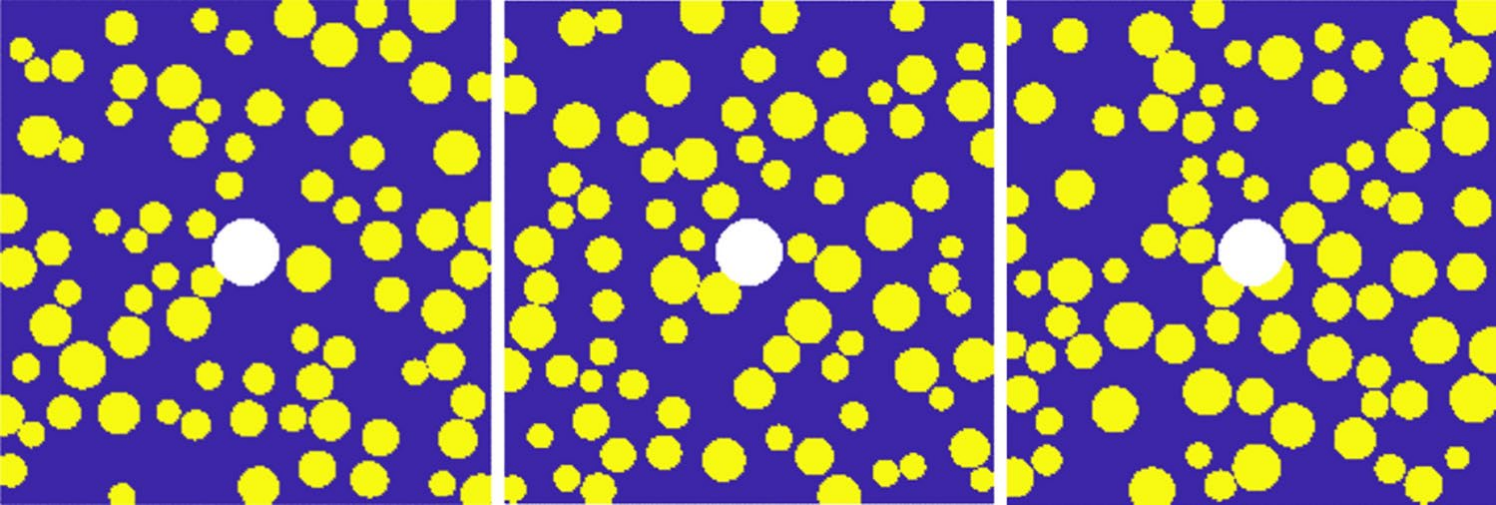
Random Aggregate Model Diagram.

For example, a random aggregate concrete model with a gradation of 4.8 mm-10 mm is generated, as shown in Fig. 5.

## Boundary damage ratio (BDR)

### The formula of boundary damage ratio

In concrete, coarse aggregates, often pebbles or crushed stones, are essential components of high-strength concrete and are not easily damaged. Investigating how the position and size of these aggregates affect the propagation of cracks in concrete is a challenging task. Traditional methods usually study the crack propagation in concrete with aggregates at fixed positions, but they fall short when it comes to studying crack propagation in concrete with aggregates at uncertain positions. To address these limitations, this paper, based on PD theory and the concept of Material Point Failure Probability Factor (PFP)^25^, introduces the novel BDR to quantitatively describe the influence of aggregates on crack propagation during concrete failure.

As shown in Eq. (8), crack propagation in PD theory can be described using the factor $\varphi (x,t)$. When the initial value of $\varphi (x,t)$ is 0, it indicates that no damage occurs at the material point $x$. When $\varphi (x,t)$ is greater than 0, it indicates that damage has occurred at the material point $x$. In the random aggregate model used in this study, due to the high strength of the aggregates, any damaged material point will consist of either the aggregate-cement interface or the cement mortar.

**Fig. 6:**
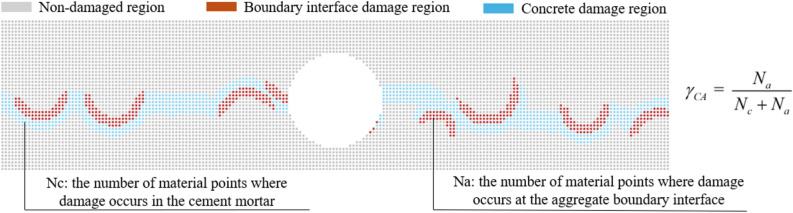
Calculation rules for $\gamma_{CA}$ under a single sample.

Therefore, the BDR can be calculated using the following equation:

12 $$\gamma_{CAi} { = }\frac{1}{{n_{i} }}\sum\limits_{k = 1}^{n} {\frac{{N_{a} }}{{N_{c} + N_{a} }}}$$

where: $\gamma_{CAi}$ represents the BDR for the gradation $i$, and $n_{i}$ represents the total number of samples calculated for the gradation $i$ using large samples.$k$ = (1,⋯, $n$).In the random aggregate concrete model, $N_{a}$ represents the number of material points where damage occurs at the aggregate boundary interface when the concrete is completely fractured. Similarly, $N_{c}$ represents the number of material points where damage occurs in the cement mortar when the concrete is completely fractured. The calculation method of $\gamma_{CA}$ under a single sample is shown in Fig. 6.

Through Eq. (12), the ratio of the total number of material points destroyed at the aggregate boundary to the total number of material points destroyed as a whole when the random aggregate model of concrete is completely destroyed under a certain level of mixing conditions can be calculated, i.e., the $\gamma_{CA}$. And in the destruction of the random aggregate model of concrete, due to the difference in the strength of the different materials, there is only the destruction at the boundary of the aggregate material points, and it is inevitable to drive the cement mortar material point damage, i.e., the $\gamma_{CA}$ < 1.

### Peridynamics method based on Monte Carlo simulation

**Fig. 7:**
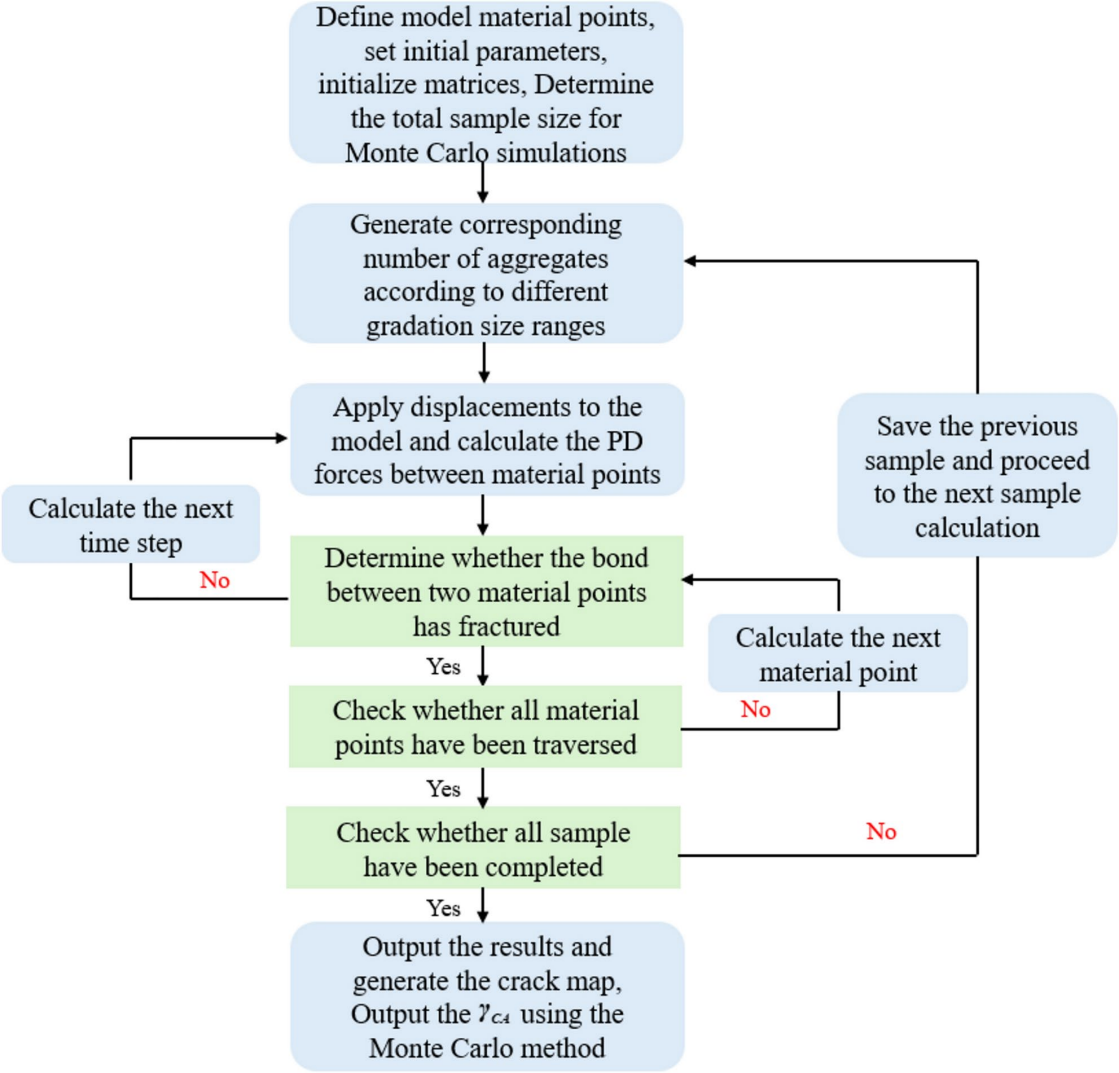
Monte Carlo Process Flowchart Based on PD Method.

The Monte Carlo method, also known as the statistical simulation method, is a numerical simulation approach based on probability and statistical theory. For complex problems such as investigating the influence of aggregates on concrete crack propagation, which are difficult to solve analytically, the Monte Carlo method proves to be an effective approach. The specific application steps in this paper are as follows:

1. Initialize the matrix and parameters in Fig. 4, generate material points in the random aggregate concrete model, and determine the horizon $\delta$, boundary conditions, and other specifications in the PD theory.
2. Determine Monte Carlo random numbers based on the involved gradations.
3. Use the PD method to calculate the crack propagation and material point displacement in the concrete model, and compute the values for different gradations under large sample sizes. The algorithm flowchart in this paper is shown in Fig. 7.

## Results and discussion

### Method validation

**Fig. 8:**
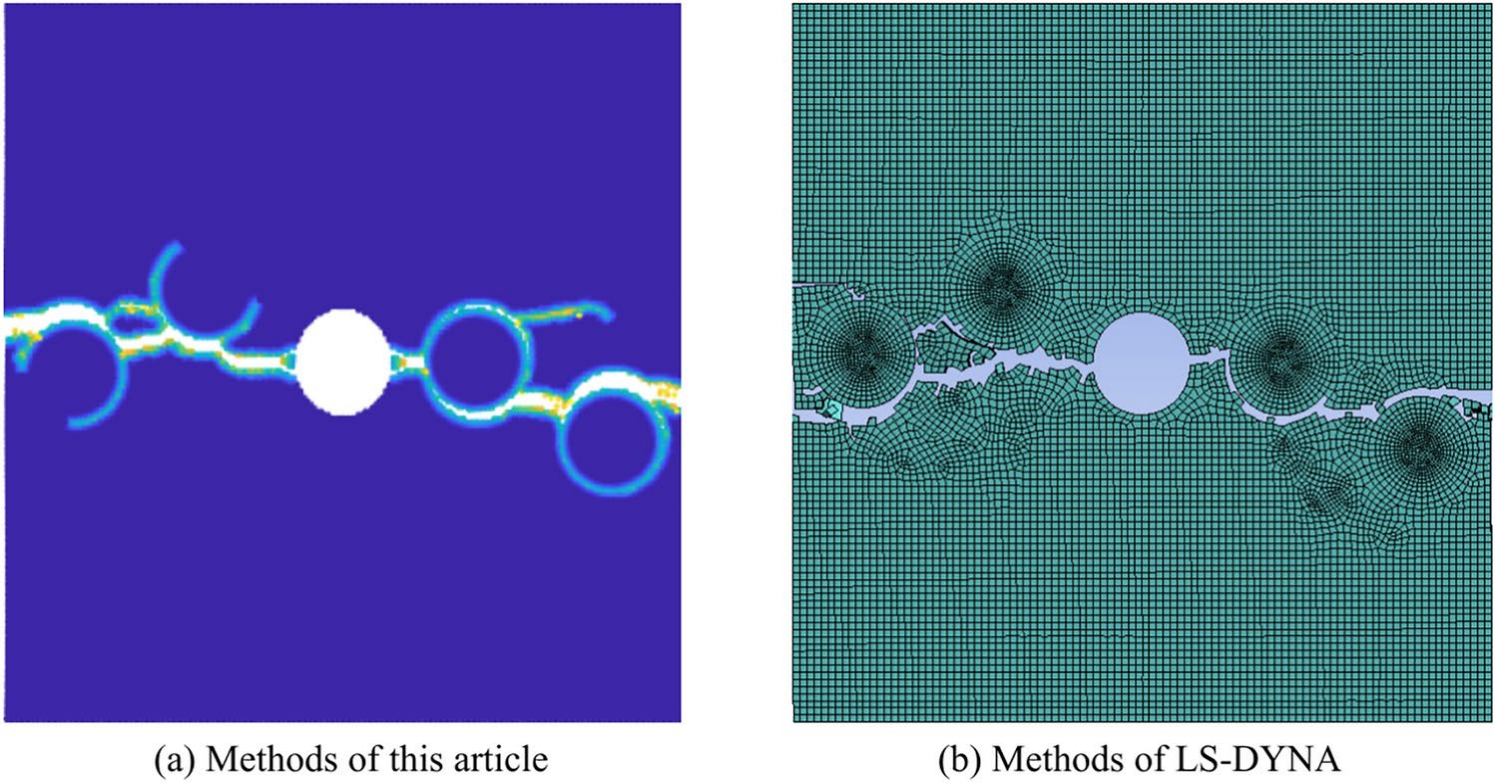
Comparison between the proposed method and the LS-DYNA method.

In order to verify the validity of this approach, a comparative validation was carried out using the finite element software LS-DYNA R8.0.0 (https://lsdyna.ansys.com). A 100 × 100 mm two-dimensional concrete model was constructed with LS-DYNA software, featuring a pre-set circular hole with a diameter of 14 mm in the center and four circular aggregates with a diameter of 15 mm placed at different positions. The model includes three materials: cement mortar, aggregate, and the interface between them. The parameters such as density, elastic modulus, Poisson’s ratio, and critical stretch rate were set to be consistent with those listed in Table 1 of this paper. The comparison results between this method and the finite element software are shown in Fig. 8, demonstrating that the crack propagation results are essentially consistent, thereby effectively validating the correctness of this method.

**Fig. 9:**
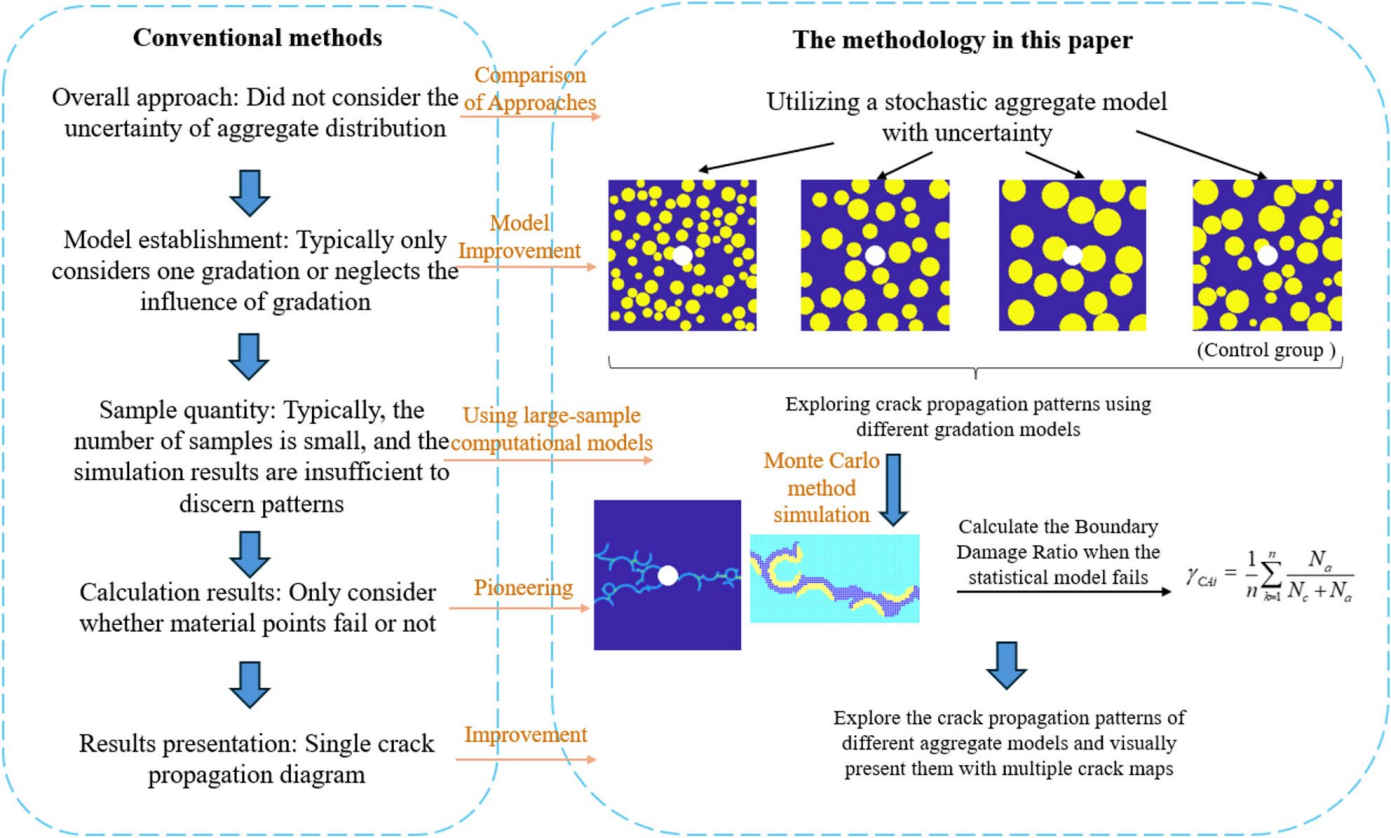
Comparison between the Proposed Method and Traditional Methods.

By comparing with traditional deterministic methods, the effectiveness of this computational method has been demonstrated. Considering the uncertainty in aggregate gradation and distribution, this method has significant advantages over traditional methods, as shown in Fig. 9.

### Results and discussion

**Fig. 10:**
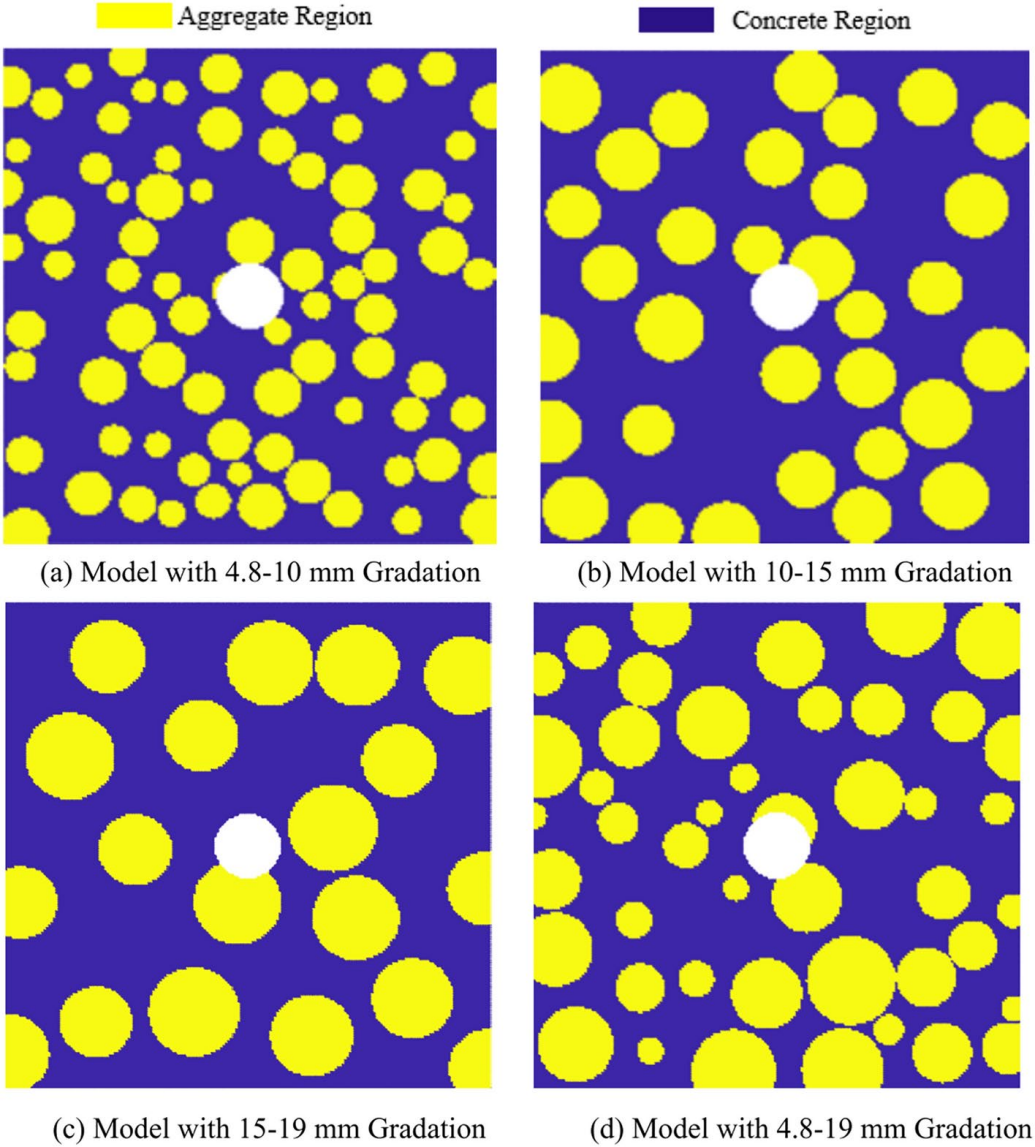
Random Aggregate Models under Four Different Gradations.

As shown in Fig. 9, the PD method was used to simulate the uniaxial tensile failure behavior of concrete random aggregate models under four different gradations. A tensile crack propagation scenario was simulated for an aggregate-containing concrete model with impurities present in a certain cross-section (the white area in the model in Fig. 10). The model setup is shown in Fig. 10. The parameter settings are consistent with the initial settings and those described in section “Random aggregate model parameter definition”. This study investigates the crack propagation process and patterns of concrete tensile fracture failure behavior under different gradation conditions.

#### The influence of aggregates on concrete crack propagation

**Fig. 11:**
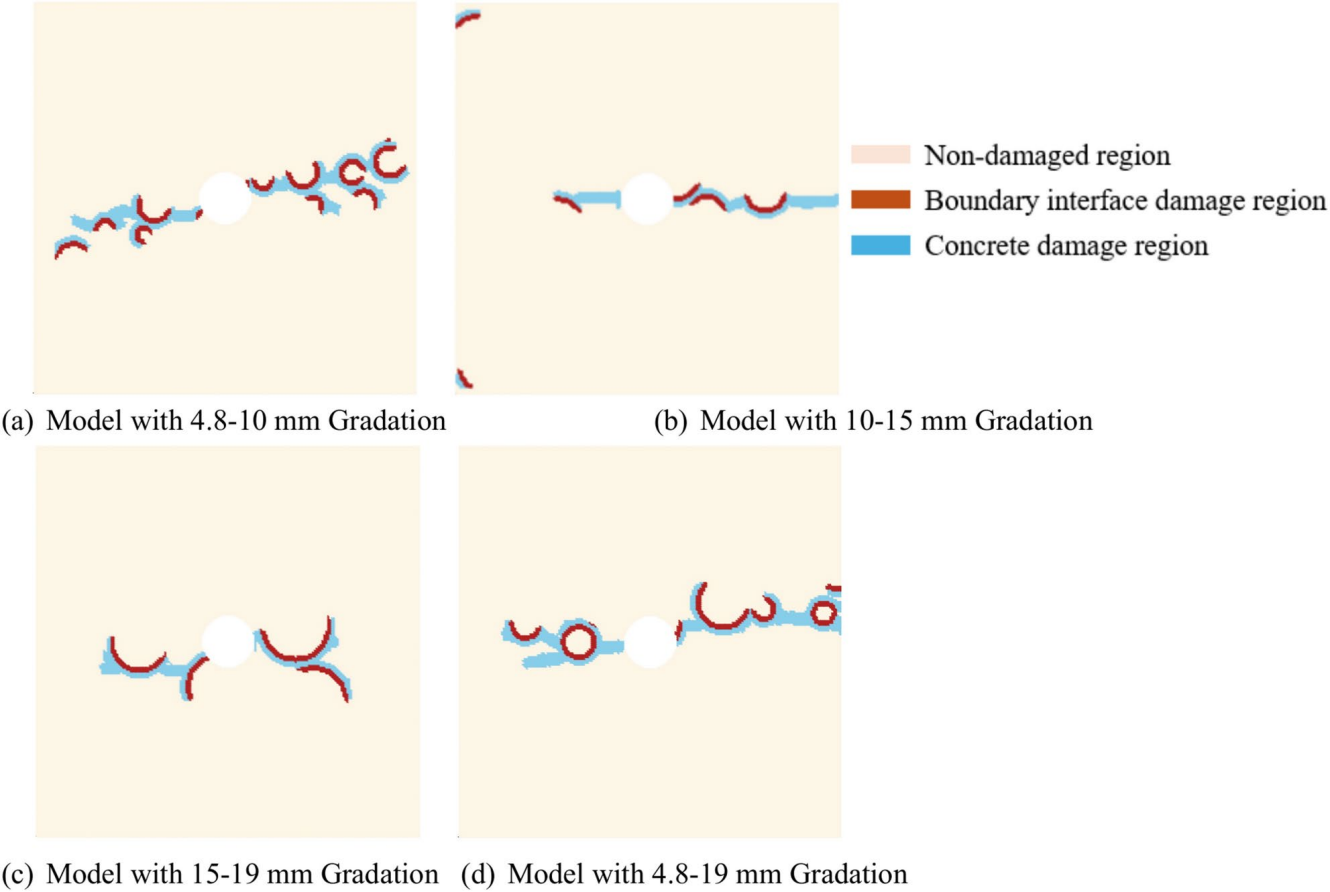
Crack propagation cloud maps at 1200 time steps for four different gradations.

To study the influence of aggregate on concrete crack propagation, uniaxial tensile fracture simulations were conducted on models with four different gradations. The adaptive dynamic relaxation method is employed, with a time step size of 1 and a total of 2200 time steps for a single sample. The results are shown in Fig. 11. When the time step is 1200, each gradation begins to experience damage, and by the time step 2200, each gradation is fully damaged. When there are impurities in the central region of the aggregate-containing concrete model, stress concentration occurs in the central region, initiating damage there, accompanied by crack formation.Comparing Fig. 10a with Fig. 11a, Fig. 10b with Fig. 11b, and Fig. 10d with Fig. 11d, it is evident that due to the weak bonding between the aggregate and concrete, cracks propagate rapidly along the aggregates near the central region during model failure, causing damage to the cement mortar material around the aggregate edges. In contrast, comparing Fig. 10c with Fig. 11c, when there are no aggregates in the small central region of the model, i.e., only cement mortar damage occurs, the failure rate is significantly slower than in the other three gradation models, and fewer cracks are produced.

**Fig. 12:**
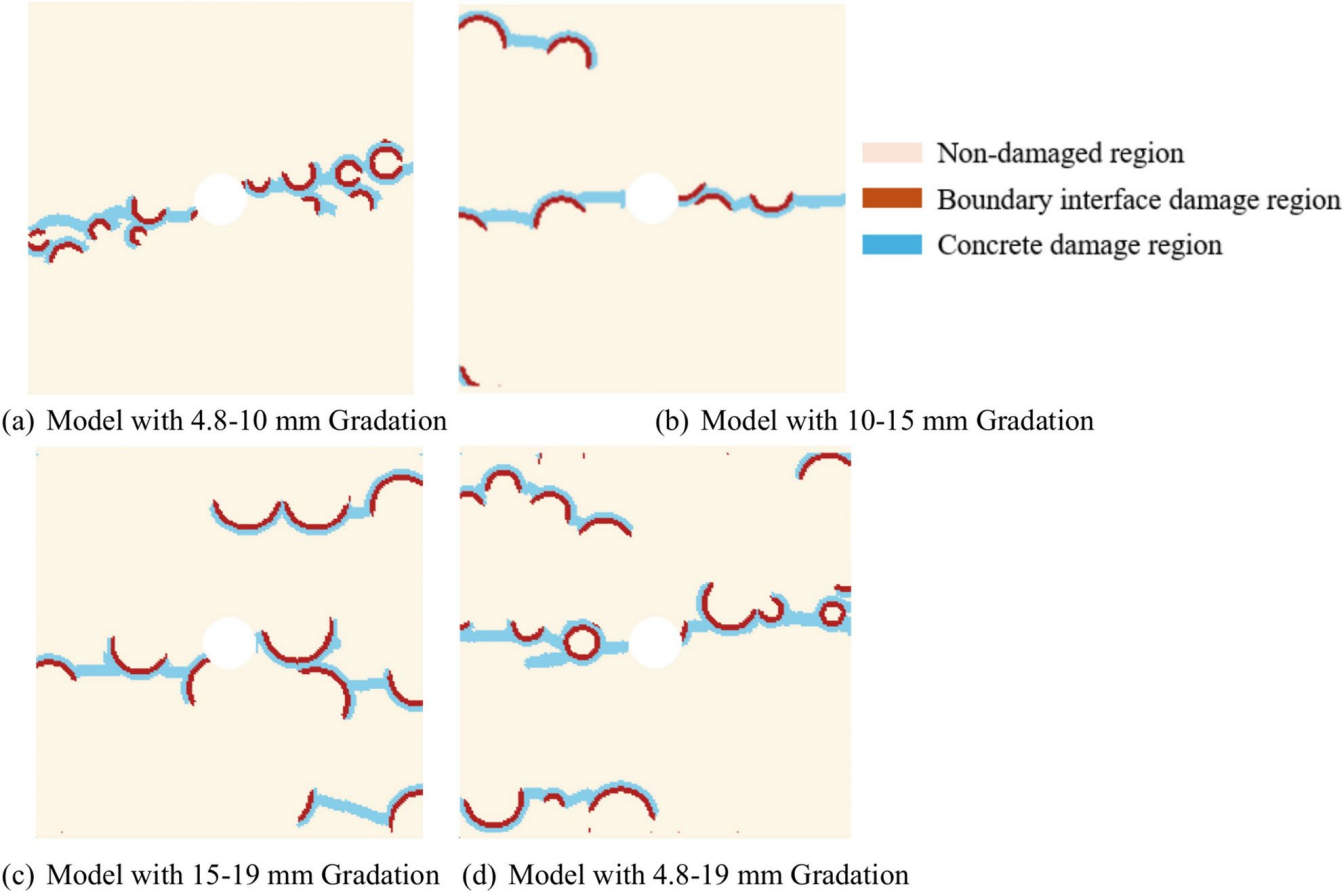
Crack propagation cloud maps at 2200 time steps for four different gradations.

According to Fig. 12, at 2200 time steps, all gradation models have been completely damaged. The aggregates in each gradation play a guiding role in crack propagation. After the model is damaged, the cracks rapidly propagate along the boundaries of the aggregates, leading to the failure of the cement mortar until the model is completely damaged.

#### The influence of aggregates on concrete crack propagation in different gradations

**Fig. 13:**
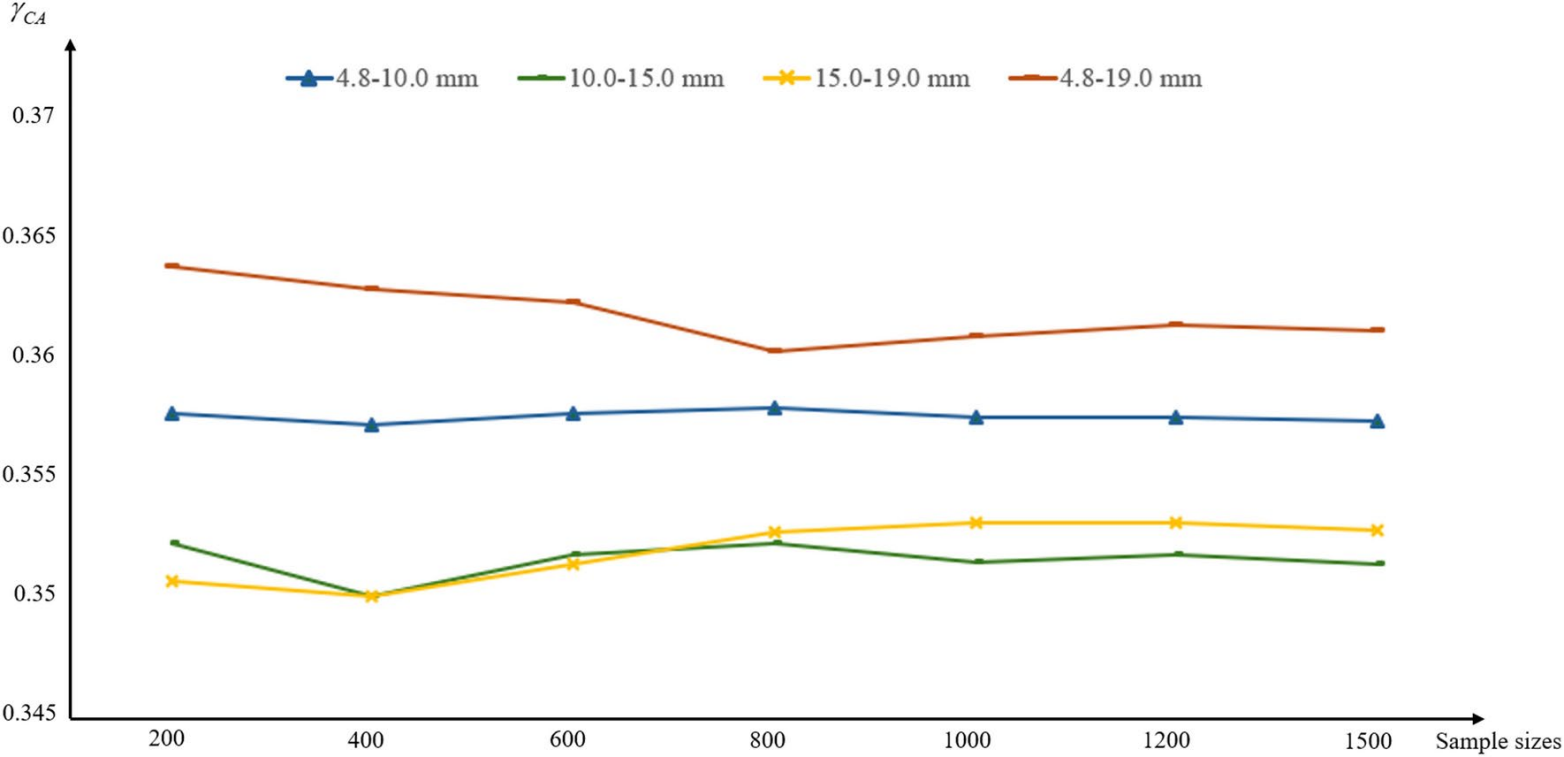
Mean of the BDR under a sample size of 1500 in the Monte Carlo method.

To further study the impact of aggregates on concrete crack propagation, Monte Carlo simulations were conducted for different gradation conditions. With a sample size of 1500, the results for the four gradation groups stabilized, as shown in Fig. 13.

The results of $N_{a}$ calculated using the PD method based on Monte Carlo simulations are shown in Table 2.

**Table 2:** Average values of $N_{a}$ different gradations under different sample sizes.

Gradation type	Average value of $N_{a}$ for 300 samples	Average value of $N_{a}$ for 600 samples	Average value of $N_{a}$ for 900 samples	Average value of $N_{a}$ for 1200 samples	Average value of $N_{a}$ for 1500 samples
4.8–10 mm	955	974	986	980	976
10–15 mm	895	922	924	927	923
15–19 mm	918	922	924	928	927
4.8–19 mm	989	991	980	982	984

The results of $N_{c}$ calculated using the PD method based on Monte Carlo simulations are shown in Table 3.

**Table 3:** Average values of $N_{c}$ different gradations under different sample sizes.

Gradation type	Average value of $N_{c}$ for 300 samples	Average value of $N_{c}$ for 600 samples	Average value of $N_{c}$ for 900 samples	Average value of $N_{c}$ for 1200 samples	Average value of $N_{c}$ for 1500 samples
4.8–10 mm	1701	1735	1753	1747	1746
10–15 mm	1645	1672	1668	1679	1675
15–19 mm	1663	1658	1652	1654	1659
4.8–19 mm	1701	1714	1702	1708	1710

According to Table 2, the total number of material points damaged at the aggregate boundary $N_{a}$ in the 10–15 mm and 15–19 mm gradation models remains relatively stable across different sample sizes, with the average value of $N_{a}$ showing no significant fluctuations and being relatively small. This indicates that the influence of aggregates in the 10–15 mm and 15–19 mm gradations is very stable, and the random distribution of aggregates in the model has a minimal impact on crack propagation. In contrast, the $N_{a}$ value in the 4.8–10 mm gradation is significantly higher than in the 10–15 mm and 15–19 mm gradations, and the fluctuations are more pronounced.

According to Table 3, the total number of damaged material points in the cement mortar $N_{c}$ is the lowest in the 15–19 mm gradation model under uniaxial tensile failure, followed by the 10–15 mm gradation, while the 4.8–19 mm gradation has the highest $N_{c}$ value after 1500 sample calculations. The $\gamma_{CA}$ was calculated according to Eq. (12), and the results are shown in Table 4.

**Table 4:** BDR $\gamma_{CA}$ Calculated for Different Sample Sizes.

Gradation type	Average value of $\gamma_{CA}$ for 300 samples	Average value of $\gamma_{CA}$ for 600 samples	Average value of $\gamma_{CA}$ for 900 samples	Average value of $\gamma_{CA}$ for 1200 samples	Average value of $\gamma_{CA}$ for 1500 samples
4.8–10 mm	0.3576	0.3579	0.3581	0.3577	0.3568
10–15 mm	0.3492	0.3519	0.3530	0.3523	0.3515
15–19 mm	0.3494	0.3515	0.3528	0.3533	0.3530
4.8–19 mm	0.3639	0.3626	0.3615	0.3616	0.3614

It can be seen that the $\gamma_{CA}$ value for the 4.8–10 mm gradation is relatively larger compared to the 10–15 mm and 15–19 mm gradations, while the $\gamma_{CA}$ value for the 4.8–19 mm control group shows a significant increase. The $\gamma_{CA}$ value reflects the extent to which aggregate size and position influence crack propagation at the point of complete failure of the model. The results indicate that, with a reasonable gradation, larger aggregate sizes have a smaller effect on crack propagation. The following sections will further investigate the control effect of aggregates on crack propagation.

**Fig. 14:**
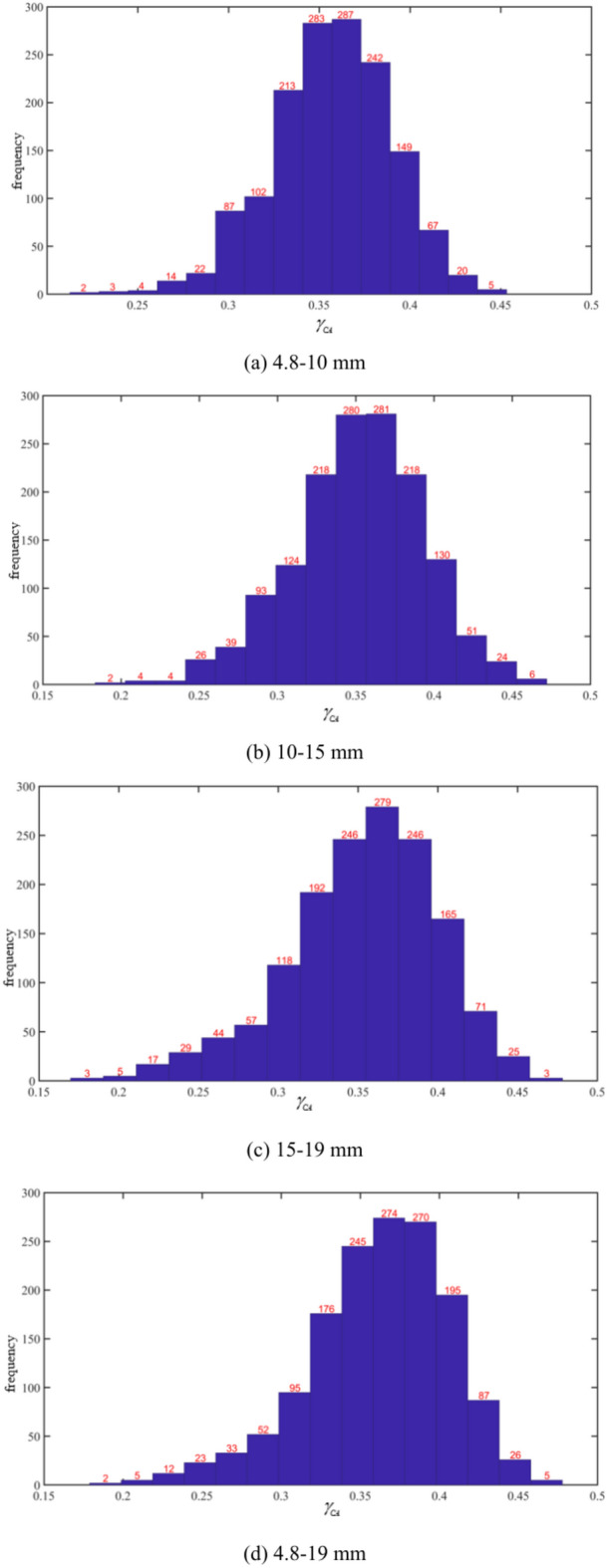
Histogram of the Frequency Distribution of BDR $\gamma_{CA}$ under the Monte Carlo Method with a Sample Size of 1500.

Based on the Monte Carlo large sample calculation of the $\gamma_{CA}$, the frequency distribution histogram is shown in Fig. 14. The results show that the $\gamma_{CA}$ values of each gradation exhibit the highest frequency near their respective mean values. Additionally, the frequency distribution histograms for each gradation show that the number of samples with $\gamma_{CA}$ values relatively smaller than the mean is significantly higher than the number of samples with larger $\gamma_{CA}$ values.

**Fig. 15:**
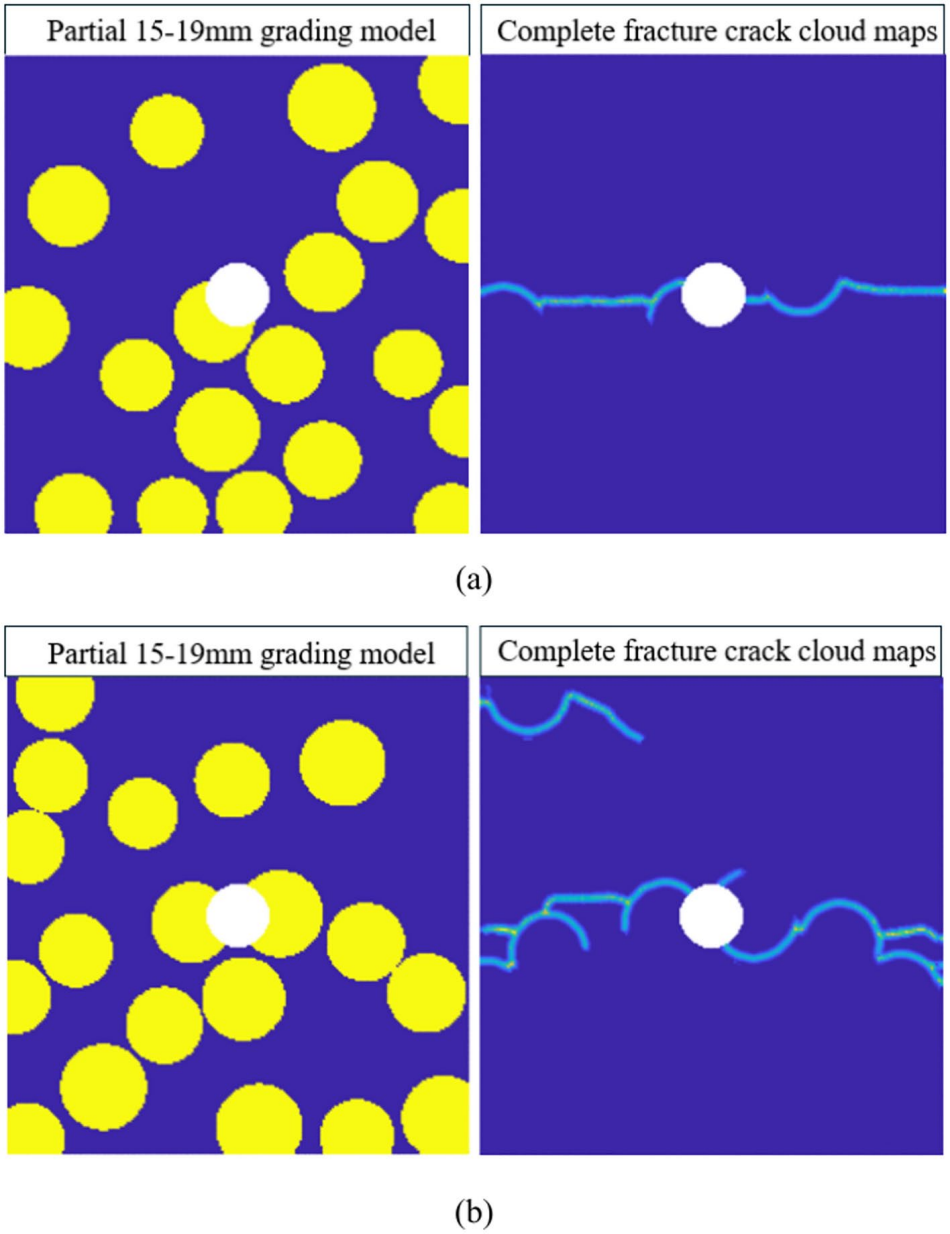
Cloud maps of complete fracture propagation in two groups of 15-19 mm gradation.

By comparing the results of any two sets from the 15–19 mm graded model, as shown in Fig. 15, it can be observed that the presence of impurities in the central region of the model causes the crack propagation direction to tend toward horizontal expansion under tensile stress. When more aggregates are present in the horizontal direction of the model’s central position, the guiding effect of the aggregates on crack propagation becomes more apparent, resulting in more cracks generated by the model’s failure. Furthermore, due to the fewer number of aggregates in the 15–19 mm graded model and their relatively uniform distribution, the crack propagation process becomes more regular, the number of interface failures remains within a predictable range, and is relatively fewer compared to other gradations. Consequently, the concrete damage induced by aggregate failure is also less.

**Fig. 16:**
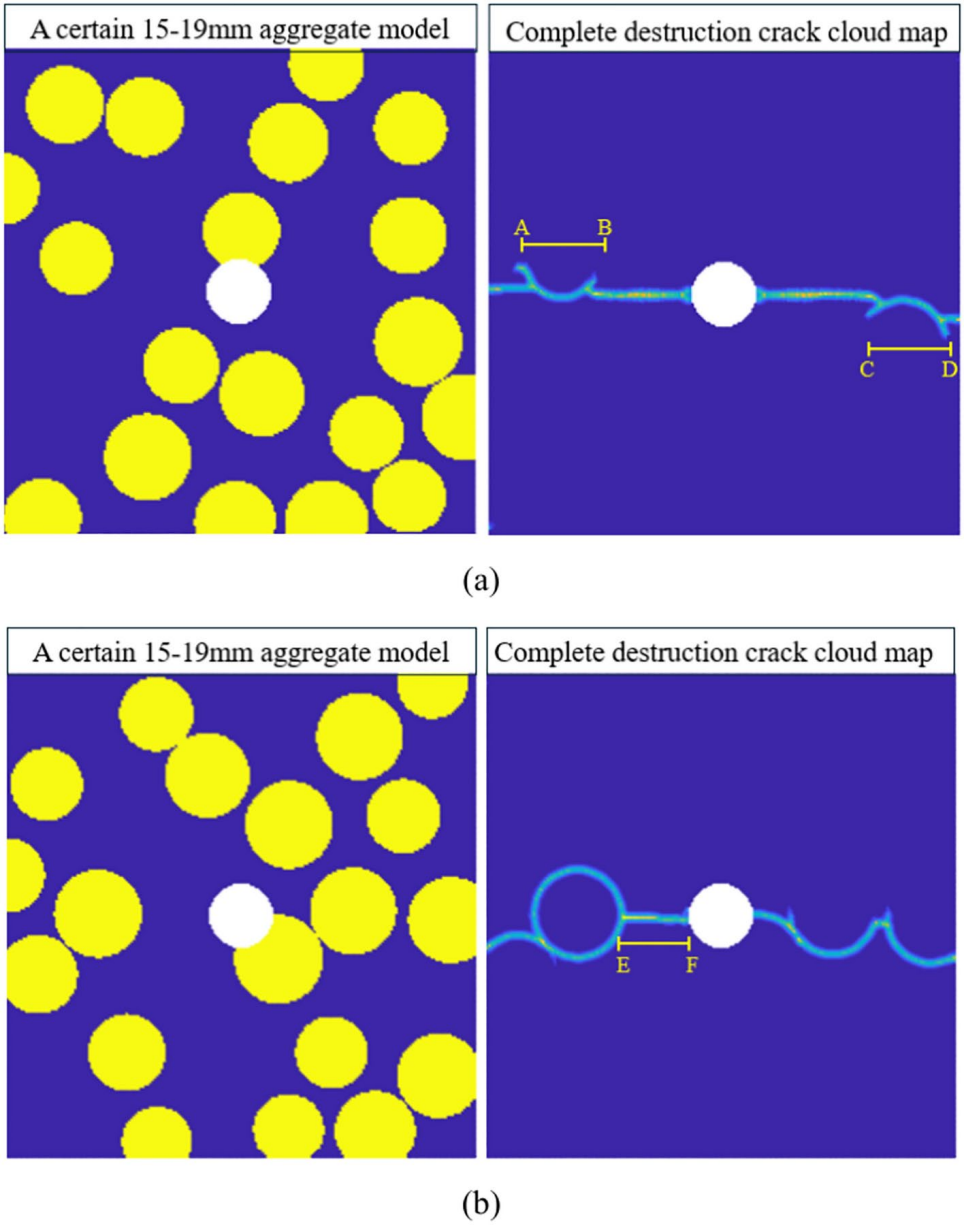
Cloud maps of complete destruction cracks under different $\gamma_{CA}$ values for two groups of 15–19 mm gradation.

As shown in Fig. 16, comparing two sets of 15–19 mm graded models with significantly different $\gamma_{CA}$ values and their crack contour maps, we see that in Fig. 16a $\gamma_{CA}$ is 0.1849, while in Fig. 16b $\gamma_{CA}$ is 0.4468. The results indicate that in Fig. 16a, the crack propagation is less influenced by the aggregates, with only two boundary damages at range AB and CD. Conversely, in Fig. 16b, the crack propagation is strongly influenced by the aggregates, with only range EF showing cement mortar damage. From the comparison in Fig. 16, it is clear that in such a model with impurities in a specific cross-section, cracks tend to rapidly propagate horizontally along the nearby aggregate-cement mortar interface after being subjected to tensile stress, continuing to expand to the next aggregate-cement mortar interface. In the same gradation, when there are more aggregates near the central impurity area or in the horizontal direction, the crack propagation path depends on the driving effect of the aggregates, causing the $\gamma_{CA}$ value to significantly increase compared to the average. Conversely, when there are fewer aggregates near the central impurity area or in the horizontal direction, the crack propagation extends in a straight line horizontally, causing the $\gamma_{CA}$ value to significantly decrease compared to the average.

**Fig. 17:**
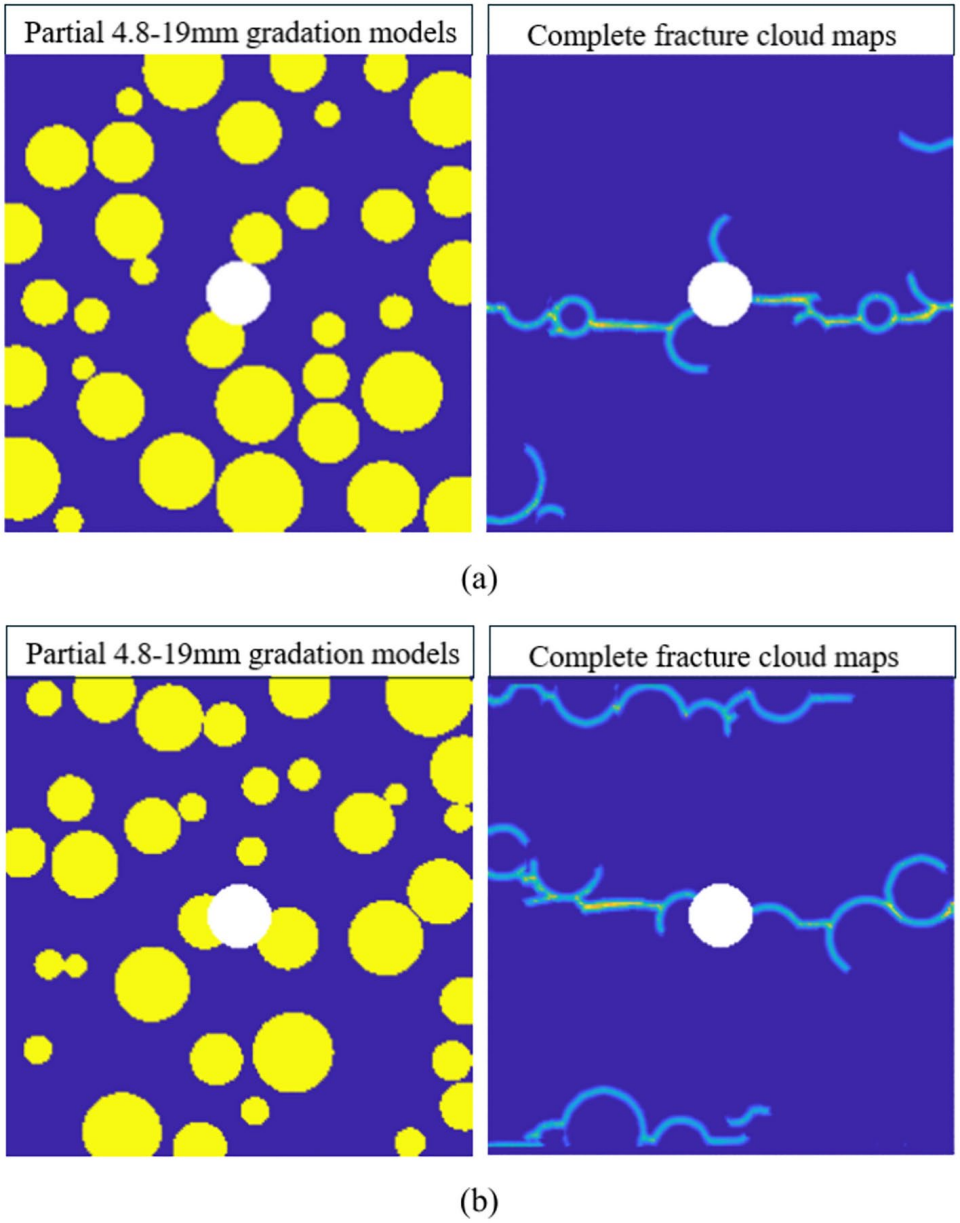
Complete fracture cloud maps for two sets of 4.8–19 mm gradation.

Comparing two sets of results from the 4.8–19 mm grading model control group, as shown in Fig. 17, it can be seen that when the number of aggregates in the model increases significantly, the crack propagation becomes more unpredictable during the model’s failure. Similarly, comparing Fig. 17a,b, when there are fewer aggregates in the horizontal direction at the center of different grading models, the crack propagation process in the central area is relatively similar. Figure 17b reveals that when the aggregate size disparity is too large and the aggregate positions are more concentrated in a certain model, the aggregates can cause extensive cracking in multiple locations within the model, significantly increasing the guiding effect of the aggregates, leading to a quicker total failure of the model. Therefore, in the case of poorly graded control groups, the aggregates completely dominate the formation of cracks and the subsequent concrete damage. The value of $\gamma_{CA}$ is significantly higher than in models with proper grading, and due to the differences in aggregate size and quantity, the fluctuations in $\gamma_{CA}$ across different samples are also more severe.

Therefore, when the concrete model containing impurities in the center undergoes failure under uniaxial tension, due to the weakness of the central area, the cracks tend to propagate rapidly in the horizontal direction from the impurity region. Because of the weak bond between the aggregates and the concrete at the interface, the aggregates play an important guiding role in crack propagation. In the 4.8–10 mm gradation and the control group of 4.8–19 mm gradation, where the aggregate size is small and the quantity is large, the guiding effect of the aggregates is stronger, resulting in a larger $\gamma_{CA}$ value and a faster model failure. Conversely, in the 10–15 mm and 15–19 mm gradations, the number of aggregates decreases, and the probability of aggregates near the impurity region also decreases. As a result, the guiding effect of the aggregates on crack propagation decreases, leading to a decrease in $\gamma_{CA}$ value and a slower model failure rate.

To further investigate the relationship between the $\gamma_{CA}$ value and different gradations, we fitted the histogram in Fig. 14. Based on the fitting results, the Weibull distribution conforms to the characteristics of the frequency distribution histogram. The Weibull distribution can be expressed by the following formula:

14 $$f(x) = \frac{{\mathbf{b}}}{{\mathbf{a}}}\left( {\frac{x}{{\mathbf{a}}}} \right)^{{{\mathbf{b}} - 1}} e^{{ - (x/{\mathbf{a}})^{{\mathbf{b}}} }} ,x \ge 0$$

**Fig. 18:**
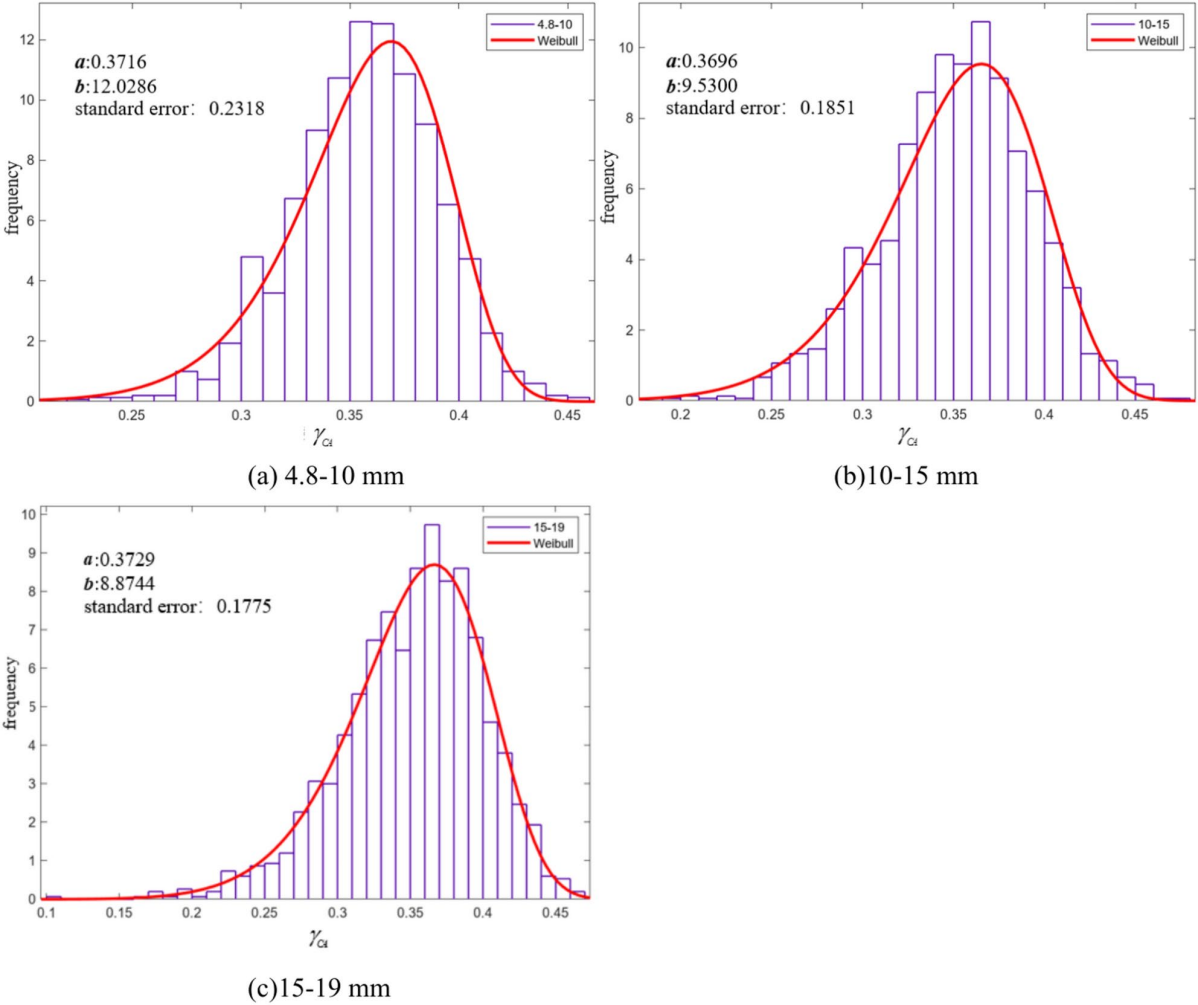
Weibull Distribution Fitting Results Graph.

In the equation, ${\mathbf{a}}$ is the scale parameter, and ${\mathbf{b}}$ is the shape parameter. The fitting results are shown in Fig. 18.

From Fig. 18, it can be seen that the standard errors for the three gradations are 0.2318, 0.1851, and 0.1775, respectively. The frequency histograms of the three benign gradations conform to the Weibull distribution. According to the fitting results, the larger the gradation size, the smaller the shape parameter in the Weibull distribution, and the more stable the graph becomes. The results indicate that the crack propagation results are most stable under the 10–15 mm and 15–19 mm gradations, with the highest frequency of $\gamma_{CA}$ around the mean value of 0.35. For the 4.8–10 mm gradation, due to the higher number of aggregates, the influence of the aggregates on crack propagation increases significantly, and the stability of the mean in the histogram distribution further decreases compared to the 10–15 mm and 15–19 mm gradations. For the control group with a 4.8–19 mm gradation, $\gamma_{CA}$ is the largest, and the influence of aggregates on crack propagation is the greatest. The frequency distribution in the histogram clearly shows that this group has the poorest stability.

## Conclusion

The present study utilizes the PD method, as outlined in Monte Carlo simulations, to examine the impact of randomly dispersed aggregates on the propagation of cracks in concrete with a central void impurity under varying gradations. The following conclusions were derived from the study:

1. A stochastic PD analysis method for concrete crack propagation that considers the random distribution characteristics of aggregates is proposed. By assigning random sizes and positions to the aggregates, the failure and damage paths of concrete can be more comprehensively represented.
2. The "Material Point Boundary Damage Ratio (BDR)" is a novel concept that has been introduced to quantitatively analyze the influence of cement mortar and coarse aggregates on crack propagation during concrete failure. This approach offers a novel methodology for examining the uncertainty and crack propagation in concrete with random aggregates. Utilizing the Monte Carlo simulation PD method, frequency histograms were plotted for substantial samples, and the fitting outcomes were found to align with the Weibull distribution.
3. A highly efficient parallel computing program for large Monte Carlo samples has been developed, enabling the study of concrete crack propagation patterns under uncertain aggregate conditions. This program has been demonstrated to achieve high computational accuracy while concomitantly enhancing efficiency.
4. Due to the weak adhesion between aggregates and cement mortar, cracks extend continuously along the edges of nearby aggregates during concrete failure. The location of the aggregates determines the crack propagation path. Concrete failure begins in the weak regions of the concrete (such as the central impurity area in this study). When aggregates are near these weak areas, the crack propagation speed increases, rapidly extending to the boundaries of other nearby aggregates. Additionally, both overly large or small aggregate sizes enhance the guiding effect of aggregates on crack propagation. Smaller aggregate sizes increase the number of aggregates, leading to a dense model where cracks consistently develop along the aggregates. Larger aggregate sizes increase the circumference of the aggregates in the cross-section, lengthening the guiding distance and intensifying the guiding effect on crack propagation.
5. Due to the high strength of the aggregates themselves, concrete containing aggregates meets the material strength requirements and enhances stability. However, the weak adhesion between aggregates and cement mortar also causes crack propagation in concrete to be more severe under the guidance of aggregates. Improving the adhesion between cement mortar and aggregates is an important issue for future research.

## Data Availability

The datasets generated during and analysed during the current study are available from the corresponding author on reasonable request.
